# Experiences from a community advisory Board in the Implementation of early access to ART for all in Eswatini: a qualitative study

**DOI:** 10.1186/s12910-019-0384-8

**Published:** 2019-07-16

**Authors:** Charmaine Khudzie Mlambo, Eva Vernooij, Roos Geut, Eliane Vrolings, Buyisile Shongwe, Saima Jiwan, Yvette Fleming, Gavin Khumalo

**Affiliations:** 1 Clinton Health Access Initiative, Mbabane, Swaziland; 2 Department of Anthropology, Amsterdam Institute for Social Science Research (AISSR), University of Amsterdam, Amsterdam, the Netherlands; 3 Aidsfonds, Amsterdam, the Netherlands; 4 Swaziland National Network of People Living with HIV/AIDS, Mbabane, Swaziland; 5 Global Network of People Living with HIV, Amsterdam, the Netherlands

**Keywords:** Community Advisory Board, CAB, HIV, Swaziland, Eswatini, Test and Treat, Early ART, Community Engagement, Community System Strengthening, Implementation research

## Abstract

**Background:**

Engaging communities in community-based health research is increasingly being adopted in low- and middle-income countries. The use of community advisory boards (CABs) is one method of practicing community involvement in health research. To date, few studies provide in-depth accounts of the strategies that CAB members use to practice community engagement. We assessed the perspectives, experiences and practices of the first local CAB in Eswatini (formerly known as Swaziland), which was implemented as part of the MaxART Early Access to ART for All study.

**Methods:**

Trained Swazi research assistants conducted two focus group discussions and 13 semi-structured interviews with CAB members who had been part of the MaxART study for at least 2.5 years. Interviews explored CAB composition and recruitment, the activities of CAB members, the mechanisms used to engage with communities and the challenges they faced in their role.

**Results:**

The MaxART CAB played an active role in the implementation of the Early Access to Art for All study, and activities mainly focused on: (1) promoting ethical conduct, in particular privacy, consent and confidentiality; (2) communication and education, communicating about the study and educating the community on the benefits of HIV testing and early access to HIV treatment; and (3) liaising between the community and the research team. Strategies for interacting with communities were varied and included attending general community meetings, visiting health facilities and visiting public places such as cattle dipping tanks, buses, bars and churches. Differences in the approach to community engagement between CAB members living in the study areas and those residing outside were identified.

**Conclusion:**

The experiences of the first CAB in Eswatini demonstrate that community engagement using CABs is a valuable mechanism for engaging communities in implementation studies. Considerations that could impact CAB functioning include clearly defining the scope of the CAB, addressing issues of CAB independence, the CAB budget, providing emotional support for CAB members, and providing continuous training and capacity building. These issues should be addressed during the early stages of CAB formation in order to optimize functioning.

## Background

Community engagement, defined as the process of working collaboratively with groups of people affiliated by geographic proximity, special interest or similar situations, to address issues affecting the well-being of those people [1], has become increasingly common in global health research [2]. In community-based research, the rationale for community engagement is to promote ethical conduct by ensuring that research is relevant to the community where it is being conducted, and that local views are incorporated into the research process [3, 4]. The specific benefits of engaging communities include protecting research participants by minimizing physical, psychosocial, social, economic and legal risks, strengthening the informed consent process by providing adequate information on research goals, risks and benefits [5, 6]; showing respect by listening to and addressing community perspectives, promoting partnership between the community and researchers [4]; and strengthening the acceptability and quality of research [7]. Recognizing that the ethical implications of research can affect communities has opened up international debate on the values, goals and mechanisms of meaningfully involving communities in the research process [4, 8, 9]. These discussions have in turn resulted in several guiding documents and reports which promote good ethical practice [10–12].

Community engagement can take a number of forms, and one of these approaches is the establishment of a Community Advisory Board (CAB) or Community Advisory Group (CAG). A CAB is usually comprised of individuals who represent the community targeted for research and who serve as liaisons between the research team and the community. The specific role of a CABs, as documented in the literature, includes: acting as a link between researchers and the community by establishing trusting relationships between the research team and the community [13–16]; educating community members about the research [16]; representing community concerns and priorities to the research team; providing the research team with insights into the social and cultural context within which the community operates; assisting in the development and review of research protocols, study materials and informed consent tools; promoting recruitment procedures; and disseminating study findings [17].

While evidence in the literature details advantages of CABs in strengthening community engagement, some studies have documented challenges faced by CABs. Some of the challenges include limited understanding of health research among CAB members [14]; inability to communicate scientific information and procedures [16]; monetary expectation and expectation of future employment [16]; dependence on research institutes or individual researchers for CAB finances and sustainability; a lack of authority to influence decisions concerning the research [14, 18] and a contradiction between the ethical mandate of the CAB and the CAB’s role in facilitating implementation of research [16]. These challenges often result in uncertainty about the advisory roles of CAB members and concerns that their involvement does not serve to adequately represent communities, but rather act as a superficial mechanism to adhere to donor requirements.

Two models of CAB composition that are commonly differentiated are the ‘broad community’ model that has representation from a cross-section of the community, and a ‘population-specific’ model that consists of the specific populations being targeted by the research project [19]. The term ‘community’ has varied definitions in the context of community-based research [20]. Community can to refer to: a group of people with diverse characteristics who are linked by social ties, share common perspectives and engage in joint action in geographical locations or settings [20]; a group of people with a common characteristic or illness; or a group of people residing within the immediate surroundings of a particular entity, e.g. a health facility [16]. Due to the lack of a homogeneous definition of the term ‘community’ in research, there are often contradictory assumptions amongst study team members about results and achievements of the contributions of a community in a particular study [20]. In the context of CABs, the lack of a standard definition of community brings forth the question of whether CAB members can indeed represent an entire community, and who should represent community interests in community-based health research. In this study, the CAB resembled the ‘broad community model’ as the CAB members were selected to represent a broad representation of social groups in different geographical locations.

This article explores the experiences of the first local CAB in Eswatini, which was established as part of the MaxART Early Access to ART for All (EAAA) study. Despite the advantages of CABs, as illustrated in the literature, previous community-based research conducted in Eswatini had not included CABs as a mechanism for engaging communities. This paper highlights the experiences of the MaxART CAB members by exploring how they engaged communities, their approach to operationalizing CAB roles, as well as the challenges and proposed recommendations, which could be valuable for the functioning of future CABs in the country and elsewhere.

## Methods

### Study context

The Kingdom of Eswatini is severely affected by HIV/AIDS. HIV prevalence is 27% (> 15 years), while HIV incidence is 1.36%, with approximately 7000 new cases annually [21]. As part of continued efforts to manage the HIV/AIDS epidemic, the Ministry of Health (MOH) introduced the MaxART EAAA study as a pilot. The study was designed to evaluate the acceptability, affordability, feasibility and clinical outcomes of offering early access to ART to all people living with HIV (PLHIV), as well as to inform national decision-making with regards to future HIV program planning.

The MaxART study used a randomized stepped-wedge design with control, transition and intervention phases implemented across 14 health facilities (and surrounding communities) in the Hhohho region. During the control phase, HIV positive clients were initiated on antiretroviral therapy (ART) following standard national treatment guidance (CD4 count of < 350 cells/mm^3^ / < 500 cells/mm^3^ [from 2015], or WHO disease stage 3 or 4) [22]. During the transition and intervention phases, ART was offered regardless of CD4 count or disease stage.

At the same time, a qualitative sub-study was conducted aimed at understanding the role of the CAB during the implementation of the MaxART study.

### CAB composition and functioning

The CAB had 24 volunteer members, aged 24 to 68 years (19 female; five male). CAB members were selected to represent different demographic and social groups and socio-economic characteristics in the community. In the communities where the study was being conducted, individuals who were knowledgeable about health issues, in particular HIV/AIDS, or were either leaders in the community or were active in community groups were proposed as potential members of the CAB.

Potential members were identified by community health committees, the MOH and the Swaziland National Network of People living with HIV/AIDS (SWANNEPHA). All CAB members received invitation letters to join the CAB, and provided either written or verbal consent to participate. Thirteen members resided in the communities where the MaxART study was conducted and the remaining eleven resided outside the study areas. The CAB represented various groups in the community, including PLHIV, youth, key populations traditional leaders, traditional healers, religious leaders, academia, nurses and community health workers.

A CAB secretary, employed by SWANNEPHA, facilitated the CAB’s formation and administrative functioning and responsibilities, included scheduling CAB meetings, taking meeting minutes, reporting and arranging CAB transport logistics. At the CAB’s inception, the MaxART research team provided trainings on the study protocol and field standard operating procedures, research ethics, the basics of HIV, the history and functions of CABs and interpersonal communication skills. Since this was the first CAB in the country, the research team also collaborated with the AIDS Rights Alliance of Southern Africa (ARASA) to conduct trainings on human rights. Six refresher trainings were provided throughout the 3 years of implementation. Other support provided included transport reimbursement for attending meetings, t-shirts and name badges (to facilitate identification during visits to study facilities) and transportation to facilities for site-visits.

Prior to the CAB’s formation, the research team contacted other teams in the African region with experience working with CABs in order to learn from their experiences, which was used to draft a terms of reference (ToR). The ToR was later discussed and finalized by the CAB members during their initial orientation session.

The CAB’s aim was to ensure that all aspects of the study were conducted in accordance with human rights and ethical conduct as stated in the study protocol. The intended role of the CAB as detailed in the final TOR included:

- providing substantive input into all aspects of the study
- serving as the voice of the community and study participants
- ensuring that the study meets local needs and contributes to improved outcomes for people living with HIV
- building community capacity in research
- properly communicating information on the study and liaising with the community
- providing the study team with recommendations on study implementation
- monitoring progress and ensuring dissemination of study findings and providing quarterly updates to the study investigators
- ensuring that the study is conducted in accordance with human rights and ethical standards

Initially, CAB members used the broad guidance detailed in the TOR to facilitate engagement with community leaders. Following observations made during the first two quarterly CAB meetings, which showed varied interpretation of the CAB’s scope and activities amongst CAB members, discussions were held between the study team and CAB members regarding key messaging on the purpose of the study and the role of the CAB. These discussions resulted in CAB refresher training sessions that focused on topics such as the CAB’s role in the community, including guidance on key topics of discussion with community members that could provide insight to the study team on community concerns about the study.

### Data collection

Of the 24 CAB members invited to participate in the FGDs, eight were unable to attend due to logistical reasons. Participants for the semi-structured interviews were selected from the list of CAB members based on their geographical location and gender. Data was collected through two focus group discussions (FGDs) and 13 semi-structured interviews with CAB members. In total researchers interviewed 16/24 (67%) CAB members and 10 of these respondents also participated in focus group discussions. The characteristics of the participants are detailed in Table 1. The FGDs covered a broad range of topics, including CAB membership and recruitment, core activities, community engagement processes and related challenges. Key issues raised in the FGDs were subsequently investigated in semi-structured individual interviews with 13 CAB members. Informed consent procedures were followed for both research components, including signed informed consent for the semi-structured interviews and verbal consent from the FGD participants.

**Table 1:** Characteristics of CAB members interviewed for the study

CAB Member Identification	Gender	Age	Residing in study area (Yes/No	Participated in FGD1	Participated in FGD2	Participated in semi-structured interviews
CAB member A	F	38	Yes	x		
CAB member B	F	36	Yes	x		x
CAB member C	F	31	Yes	x		x
CAB member D	F	41	No	x		x
CAB member E	M	42	Yes	x		x
CAB member F	F	53	Yes	x		x
CAB member G	M	31	Yes	x		x
CAB member H	F	31	Yes		x	x
CAB member I	F	33	Yes		x	x
CAB member J	F	38	Yes		x	
CAB member K	F	56	No		x	x
CAB member L	F	59	No		x	
CAB member M	F	29	No		x	x
CAB member N	F	28	No		x	
CAB member O	F	39	Yes		x	
CAB member P	M	58	Yes		x	
CAB member Q	F	31	Yes			x
CAB member R	M	32	No			x
CAB member S	F	54	No			x

Swazi research assistants conducted the interviews in either Siswati or in a mix of English and Siswati. All interviews were recorded with digital voice recorders. Afterwards, the interviews were transcribed word for word and translated into English by the research assistant. The Swaziland National Health Research Review Board granted ethical approval prior to the study’s commencement (Ref: MH599C/IRB 0009688/NHRRB 027/16).

### Data analysis

Data were coded manually and thematic content analysis was utilized to pinpoint the central themes. Preliminary codes were based on initial research questions, and other significant topics that were identified during the first review of the interview transcripts were then included. KM carried out line-by-line coding in collaboration with GK and BS, who also discussed preliminary conclusions.

## Results

### Perceived role of the CAB

The intended role of the CAB in the EAAA study, as stated in the study protocol, was to “ensure that all aspects of the study were conducted in accordance with human rights and ethical standards” [23]. This broad and ambitious task was further detailed in the CAB TOR to include functions such as communicating information on the study, liaising with the community, serving as the voice of the community and providing the study team with recommendations on study implementation. In addition to the information in the TOR, the study team also utilized sessions during CAB refresher trainings to re-cap on the intended roles.

CAB members interviewed in this study understood their role as fulfilling three main functions: (1) promoting research-related ethical conduct, in particular privacy, consent and confidentiality to community members; (2) communicating about the study and educating on the benefits of HIV testing and early access to HIV treatment; and (3) liaising between the community and the study team. Although these roles mostly represented the broad guidance provided in the CAB terms of reference, as well as the messages used by the study team during CAB trainings, some variations were noted in the interviews with regards to details in the mechanisms employed by CAB members and the interpretation of their role when engaging community members. These issues are discussed further below.

#### Promoting ethical conduct

Nearly all CAB members mentioned their main role was to ensure that the human rights of community members were protected during the research process. In line with the guidance from the study team, the CAB’s interpretation of this role included examining issues of privacy, consent and confidentiality for HIV positive study participants; enquiring whether study participants were informed of the study prior to enrollment and that informed consent was provided; enquiring into issues of coercion/forced ART initiation; observing that quality health services were being provided in the health facilities; and observing health worker well-being and working conditions in facilities. Topics discussed with clients and health workers in relation to this role included how the MaxART study was communicated to study participants, the relationship between health workers and the study team and adherence to informed consent procedures.

In addition to providing operational guidance on promoting human rights and ethical standards, the study team also recommended ways in which CAB members could engage with the community as they executed their role. The proposed ways in which CAB members interacted with the community were varied, but mainly constituted observing and listening, as well as talking to community members. The CAB members who were residing in the study communities were advised to informally interact with community members in order to gauge perceptions about the study. As a result, observing the waiting areas of study facilities and listening to people were the main approaches to gather insight into the study implementation. CAB members approached facility drop-ins and accompanied clients to educational health talks. Their methods also included joining patients in health facility queues, monitoring communication between health workers and clients and chatting informally with clients so as to document their attitudes towards and experiences of early ART initiation, the overall quality of service delivery at the facility and gaps in service delivery. In some instances, they disclosed their role as a CAB member to the clients. The following quote illustrates how these techniques of observing and listening were successful methods to gather unbiased information in a non-intrusive manner:

*“Ok, my strategies which I thought worked better and perfect don’t come as a CAB member but as a community member. Be a client also, come as a human being, there is nothing different about you anyways when you are a CAB member. So, when you come, sometimes there is even no need to ask questions, people will be talking about these issues when standing in the queue waiting for a bus in the morning, about EAAA or even ART. So try to probe some few questions, try to engage like a client. Take out your diary and start writing some few notes that you have discovered. Or else as some point you can even engage one client and explain to them that you are a CAB member and I would love to engage you on questions on the MaxART program that is going on here and if you don’t mind. If the client agrees to that, you can proceed with asking.” (CAB member 2).*

When the CAB was initially formed, CAB members did not have any identifying clothing. Half way through the implementation of the MaxART study, as a result of recommendations from a case study on the functioning of the CAB [24], CAB members were provided with t-shirts and picture name tags to wear during health facility visits. The case study highlighted that CAB members who did not disclose their role while actively seeking information from community members could potentially violate research ethics. CAB members were therefore advised by the study team to disclose to people their role as CAB members during informal conversations.

For CAB members who did not reside in the study areas, visits to health facilities were part of pre-scheduled visits. These visits were usually attended by the CAB secretary, the CAB chair or co-chair and an additional CAB member. For these visits, the CAB secretary obtained permission from the facility management for CAB members to engage with health workers and clients in order to understand how the study was being implemented and obtain information on concerns and recommendations.

#### Communication and education

According to the CAB TOR, another CAB role was communicating information about the purpose of the study, the benefits of early ART initiation, the study duration and the study implementation sites. In the interviews conducted with CAB members, it was evident that they fulfilled this role, including motivating people to participate in the study and debunking common myths about early ART use. They also expanded upon their initial role by including education on health issues outside the scope of the study.

CAB members advised people on general health issues and actively advocated for people in their communities to start HIV treatment early. This was partly related to the fact that some of the CAB members, such as those who were nurses, rural health motivators and community health committee members, were knowledgeable about medical issues and had prior experience talking to people about health care issues. In addition, the trainings provided to the CAB as part of the study included information on several health topics, including HIV/AIDS. Thus, CAB members did not always differentiate between their role in the CAB and their other health-related duties, but rather incorporated the CAB role and the study messages into their ongoing work.

In order to reach different members of the community, CAB participants utilized existing social structures and networks. These included bars, community meetings, cattle dipping tanks, women’s meetings, clinics, support group meetings for PLHIV, churches and public transportation. The community networks allowed for daily interaction with various community members, and since many of the CAB members were already known in the communities, this facilitated acceptance and trust from community members, as illustrated in the following quote.

*“I saw that it [the CAB] is very important, because people in the community would listen to a person who stays within the community, compared to an outsider who just comes and goes. Only few will listen to a person from outside… whereas if someone from the same community says there is this program, it helps there and there, tries and explain to them, they will understand.” (CAB member 10).*

#### Liaising between the community and the study team

In addition to the facility visits and community interactions, another strategy that enabled the CAB to address community concerns was the creation of a 24-h toll-free hotline. Previous studies had created hotlines for CAB members [7], but in this study, the hotline was also accessible to community members. The hotline’s purpose was to allow for quick reporting on study-related concerns or issues. The CAB secretary ran the hotline, which was funded through the study budget at a cost of USD 76 monthly. The hotline also received calls outside its purpose, including on: gender-based violence, missing or late laboratory results, shortage of commodities at facilities, health care worker workload, health care worker attitudes, changes in health care services (both positive and negative) at facilities and improved quality of life from clients enrolled in the program. Urgent issues received through the hotline were communicated to relevant focal persons in the study (e.g. issues concerning facilities were communicated to the study’s clinical coordinator to be addressed with management in relevant facilities).

CAB members’ interactions with community members allowed them to obtain input from the communities which they reported back to the CAB secretary and study team. These feedback mechanisms allowed the CAB to share the diverse perspectives of a broad range of people in the communities.

*I remember in one of the meetings with one of the ministry of health deputy directors she said when we explain about the experiences of the people, it gives them (the Ministry) a clear picture of what is really happening in the facilities. She said boards like CAB should be given more opportunities to voice people’s problems because they bring them to surface, things that the Ministry who have had no knowledge of… (CAB member 1).*

### Value of the CAB to CAB members

Although the study did not seek to document the reasons why people joined the CAB, the members reported that participating on the CAB had resulted in positive personal outcomes. The outcomes were categorized into: increased learning, increased community recognition and increased ability to help others. The CAB members identified CAB convenings as opportunities to gain knowledge around HIV, research ethics, issues facing the communities where they live and how to work with people from diverse backgrounds. CAB members also identified areas where they wanted to gain skills and knowledge, and trainings were provided on these topics, including on interpersonal communication and key populations (sex workers and men having sex with men).

CAB members expressed pride in being part of the CAB and the respect they received from the community, for example when traditional leaders allowed them to communicate about the study in community meetings, as is illustrated in the quote below;

*We (the CAB) are taken seriously because when you ask for a slot during meetings, they (community leaders) give you without any problem so they (community leaders) really helped us (the CAB) in letting the community know about the EAAA, they even let us know about other meetings and we would prepare ourselves. (CAB member 7).*

CAB members also took pride in the fact that their work positively affected the lives of others. The majority of CAB members were already involved in their communities, including as outreach workers and providers of peer support. Through CAB, this involvement extended to encompass treatment education and accompanying people who experienced lapses in treatment back to the clinic. CAB members also mentioned testimonies of people coming back to thank them after they had started treatment and were feeling better. They mentioned providing support to community members with addressing family-related issues. This support was either direct, by providing advice, or indirect whereby they referred the community members to other organizations within their communities who would be able to provide assistance.

*It also made us see how important we are as a CAB in Swaziland because of the part we played. Government alone couldn’t have played this role that we play alone… (Focus group participant 3).*

### Challenges in CAB role implementation

The interviews raised some issues that hindered the CAB’s ability to function. The main issues were: lack of adequate funding and dependence of the CAB on the study team for finances; structure of CAB meetings; dealing with emotional issues from community members; concerns about CAB sustainability and lack of community awareness of the CAB’s role in some places.

#### Insufficient financial resources

CAB members stated that the budget allocated for CAB functioning was insufficient. For example, the CAB did not have its own car for site visits, which made it extremely challenging to reach rural clinics in the mountainous Hhohho region, particularly in the rainy season. In terms of communication costs, CAB members also did not receive paid airtime; despite the creation of a WhatsApp group and a toll-free hotline to contact the CAB secretary, CAB members said they would have benefited from airtime to more easily communicate with each other.

#### Reliance of the CAB on the study team

The CAB relied on SWANNEPHA for several aspects of their work including: meeting coordination, CAB member communication, arranging transportation to visit clinics, travel reimbursements and training logistics. This reliance on the study team was challenging for CAB members who did not live in the geographic areas where the study occurred, as any delays on SWANNEPHA’s end in approving logistics and finances impacted the ability of these CAB members to travel and attend meetings.

#### CAB structure and functioning

The CAB members described a positive working relationship with fellow CAB members. There were logistical concerns mentioned related to the location of the meetings which were held at the SWANNEPHA offices in the city of Mbabane, which resulted in long travel distances to attend meetings. The long travel times affected the length of the CAB meetings, due to the late arrivals of some members.

During the first 18 months of study implementation, CAB meetings were held on a quarterly basis, where all CAB members met to discuss issues arising from facility and community visits as well as the hotline. During the CAB case study, it was noted that quarterly meetings were spaced too far apart to allow for the CAB to address critical study implementation issues. The frequency of the meetings was subsequently modified; in addition to the quarterly meetings for the entire CAB, bi-monthly meetings were introduced for the CAB chair/co-chair, CAB secretary and one or two CAB members to discuss emerging issues and provide recommendations to the study team. The bi-monthly meetings allowed for a faster response to urgent issues. CAB meetings usually averaged 5 hours, but this was often insufficient to cover all agenda items.

#### Dealing with emotions and issues beyond the scope of the study

CAB members found that people in their communities approached them with a range of issues, some related to the study and others beyond the scope of the study. The study team provided the CAB with a referral list of organizations available in the different study communities, so that CAB members could make referrals. In the interviews, CAB members mentioned that hearing the issues from community members could sometimes be difficult emotionally, especially since they had a role to provide support to the person confiding in them, as the following quote illustrates;

*It happens that at some point the issue becomes very difficult and you can feel it, in a way that you feel like crying, but then you are supposed to be strong for yourself and the client to keep the conversation on progress. (CAB member 8).*

#### Lack of awareness in the community

One CAB member mentioned that it was challenging to gain the trust of community members without first meeting and seeking approval from community leaders. The study’s process for introducing CAB members to local communities varied in the beginning; in some places, they met traditional leaders at the Inkhundla (constituency) while in others they met with the inner council. The Inkhundla is an administrative subdivision which includes a cluster of chiefdoms (imiphakatsi) and form the political structures through which Eswatini’s administration is organized. This limitation was highlighted in a case report and thereafter, another round of CAB introductions was done in all the study communities which facilitated better functioning of the CAB.

## Discussion

### Promoting human rights and ethical standards

The importance of engaging communities through establishing CABs when conducting community-based research has been well articulated in the literature [8–10, 14, 19, 25–27]. CABs have been linked to having a role in protecting the rights of study participants and communities, and in several studies this has meant that they have input in study design and the development of study protocols, and in reviewing informed consent forms and study tools in order to ensure that these initial steps in research design are respectful of participants and communities [15, 19, 25]. During study implementation, the selected CAB members continue to provide input and to address ethical issues [19, 26]. Since CAB members are usually either selected to represent the diverse demographics in communities or the specific communities being affected by an issue [19], their input during study implementation may help protect the interests of the community.

However, a question arise in terms of how CABs address ethical issues that arise in the community. Is the scope of CAB members limited to discussing ethical issues highlighted by research teams during CAB meetings, or do they have a more independent role in monitoring study implementation to ensure research is implemented according to ethical standards? In the literature, there some accounts of CABs that engage with people in their surrounding areas/communities in the context of sharing information about the study and helping with recruitment and retention [14, 16, 19] but not in the context of protecting ethical standards or being actively involved in field activities during study implementation. Few studies have described how CAB members extending their scope to practically monitor ethical issues in the community. For example, in a study conducted by Nyirenda et al. [16], CAB members reported accompanying researchers to participant’s homes, which was outside the scope of the CAB as defined in their study. Implementation study teams should have reflective consultations during the design phase about the possibilities and mechanisms of granting community representatives a responsibility of monitoring ethical conduct. This includes what internal mechanisms need to be in place so that the CAB is able to affect decisions regarding ethical study procedures and avoid CAB engagement that is tokenistic, as alluded to by Nyirenda et al. [16].

In the MaxART study, the CAB’s role was to promote that the study was conducted in accordance with human rights and research ethical standards. Thus, in addition to representing the diverse demographics during CAB meetings with researchers, the CAB also had a role in being the ‘eyes and ears’ in communities and in order to provide the study team with insight on its implementation. Having the CAB actively engage with community members and promote ethical aspects of privacy, consent and confidentiality added value for the research team, since they were able to have access to information from a variety of sources in the community that otherwise could not have been obtained. Some CAB members capitalized on their already established roles in the community to also use these forums to share information about the study and gather insight into people’s concerns regarding study implementation. Since they were already trusted members of the community, they were also a trusted link between the study team and community members.

In terms of the role of the CAB in the communication and education in the community, similar to a study conducted by Lwin et al. [15], the study findings showed that the role of the CAB as understood by CAB members was much broader than originally envisioned by the study team. CAB members took on additional roles as health educators, encouraging better healthcare seeking behaviour and providing healthcare advice to community members. This could be as a result of CAB members merging their CAB roles with their existing community roles. While this strategy provided a mechanism for the study messages to be shared with communities, it also had potential challenges, especially given that not all the CAB members had formal training in healthcare or health education. A recommendation as provided by in Lwin et al. would be to capitalize on the opportunity the CAB poses, and provide appropriate training and support to CAB members and to better combine their CAB role with a range of other community engagement activities [15] .

In this study, we found that the MaxART implementation study did not provide adequate guidance to the CAB about their role of ‘protecting communities’. This task was ill-defined in the initial trainings and little guidance was provided by the study team during subsequent trainings about the interpretation and boundaries of ‘protection’ CAB members should provide. When forming CABs and through-out the implementation of the research, is important that the purpose of the CAB is clear to all members [16, 27] and that the ways that they represent and interact with communities is discussed in order to identify any issues that may mitigate the functioning of the CAB.

### Lessons for future CABs in the country and elsewhere

The main aim in conducting this work was to better understand how the CAB members perceived their role, to gain insight into the on-the-ground mechanisms that they used to engage with community members and to understand the challenges they faced in their role.

As confirmed in other studies [14, 15, 19, 28], there was value in having a CAB as part of the MaxART EAAA study. CAB members had a role in communicating and sharing information about the study and sometimes dispelled misconceptions about the study in their communities. They provided feedback to the study team; acted as a link to and a voice of the communities; and advocated for the interests of study participants including issues arising around service delivery in health facilities.

While the CAB contributed to strengthening community engagement, there were some challenges highlighted by CAB members that provide important lessons for future CAB functioning, including around CAB independence, appropriate budget allocations, increasing psychosocial support for CAB members, and providing continuous training and capacity building.

The dependence of the CAB on the MaxART study team for support regarding issues such as the logistics of facilitating meetings, especially transport, was a challenge for CAB members. Other studies have recommended the use of non-study-specific CABs [6, 15], arguing that such a structure not only allows for independent functioning of the CAB, but also encourages sustainability as the relationship between the CAB and the community lasts beyond the duration of one study. In our context, a recommendation would be to establish a non-study-specific CAB, the operations and budget of which could be facilitated as an independent entity, or to explore having a CAB that is affiliated with the Ministry of Health, although this would also pose potential issues around independence.

It is essential that CAB members create a safe space for community members to share their concerns and experiences. As a result, some issues arise that are outside the study remit, including community members sharing personal issues. This places a burden on CAB members emotionally and may require expertise outside of the CAB. It is critical to provide psychosocial support to CAB members throughout the process. In the MaxART study, a referral list was provided to the CAB, but examining the effectiveness of this referral process was not part of the evaluation. Future studies could evaluate the use of this system.

Training CAB members on topics such as the study protocol, the particularities of the intervention being researched or implemented, and research ethics has been recommended in other studies [15, 16, 19]. Based on the findings of this study, we recommend that rather than only conducting orientation trainings at the beginning of the study, refresher trainings should be offered continuously throughout the period of study implementation. CAB members should be consulted regarding areas in which they require capacity building and trainings should then be customized. This recommendation has cost implications which should be carefully considered during the CAB establishment phase.

There are a few accounts in the literature that describe CAB activities in the field and how CAB members engage with people in their surrounding communities. These examples often talk about CAB members sharing information about the study and helping with recruitment and retention [14, 16, 19], but do not cover the CAB playing a role in study implementation. In the MaxART study, the CAB’s role included being actively involved in advocating for the study to be conducted in accordance with standards of ethical conduct in the study sites, as well as being the eyes and ears in communities and engaging with community members in order to provide the research team with feedback regarding possible ethical malpractices.

When forming CABs, and throughout the implementation of research, it is important that the scope of the CAB be continuously reviewed with CAB members [16, 27], and that the ways in which they represent and interact with communities be discussed, in order to identify any issues that may impact the functioning of the CAB. CAB ToRs and functions should be clearly communicated to the CAB, the research team and community members.

### Limitations of the study

This study did not aim to evaluate CAB functioning in the communities, but rather to understand the roles as perceived by CAB members and the strategies they employed in engaging communities during the MaxART EAAA study. This approach meant that the views of community members and the study team, and their experiences of working with the CAB, were not included. In order to gain a holistic understanding of the role of the CAB in community engagement and its effectiveness, it would be important to also include their perspectives. Furthermore, the inability to interview the the entire CAB membership is a limitation to the study.

## Conclusions

The experiences of the first CAB members in Eswatini demonstrate that using CABs is a valuable and insightful mechanism to engage communities in a study’s implementation. In areas where CABs are not required for community-based research, governing authorities could use these findings to advocate for the inclusion of community engagement mechanisms, and in particular the creation of CABs, in future research studies. That said, the study also highlights some considerations that could impact CAB functioning. These include the need to clearly define the scope, responsibilities and feedback mechanisms, address issues around independence, provide psychosocial support and offer continuous refresher trainings. These issues should be considered during the early stages of CAB formation to optimize CAB functioning.

## Data Availability

The datasets used during the current study are available from the corresponding author on reasonable request and with permission of the study’s principal investigator.

## Abbreviations

ARASA: AIDS Rights Alliance of Southern Africa
ART: Antiretroviral therapy
CAB: Community Advisory Board
CAG: Community Advisory Group
EAAA: Early Access to ART for All
FGD: Focus Group Discussion
MaxART: Maximizing ART for Better Health and Zero New Infections
MOH: Ministry of Health
PLHIV: People Living with HIV
SWANNEPHA: Swaziland National Network of People Living with HIV
TOR: Terms of Reference
